# Aplasia cutis congenita: Two cases of non-scalp lesions

**DOI:** 10.4103/0970-0358.59297

**Published:** 2009

**Authors:** Tarek A. Abulezz, Mahmoud A. Shalkamy

**Affiliations:** Department of Plastic Surgery, Faculty of Medicine, Sohag University, Sohag, Egypt

**Keywords:** Aplasia cutis congenita, congenital skin defect, neonatal scalp defects

## Abstract

Aplasia cutis congenita (ACC) is a rare condition characterized by a localized absence of skin and in some cases, the subcutaneous tissues. The majority of cases occur in the scalp; however, the lesion may occur anywhere in the trunk and extremities. ACC is most often an isolated defect, but it can be associated with other anomalies. Most reported cases are sporadic with a few reports of familial occurrence. Neither the pathogenesis nor the aetiology is clarified. Healing is spontaneous in most cases, and apart from keeping the lesion clean, no specific treatment is required. In this report, two cases of non-scalp ACC occurring in the lower limbs are presented and a brief review of the literature is conducted.

## INTRODUCTION

Aplasia cutis congenita (ACC) is a rare congenital disorder characterized by a localized absence of skin, dermal appendages and in some cases subcutaneous tissues. It was first described by Cordon in 1767.[1,2] ACC may occur anywhere in the body; however, in 84% of cases, the defect is found in the scalp,[3] where it is often solitary and located predominately in the midline vertex. Non-scalp lesions may involve the trunk and/or extremities and are usually bilaterally symmetric.[4] Still, asymmetric distribution has been also reported.[5] At birth, the appearance of the lesion may vary from superficial erosion to a deep ulcer with the affected area covered with a thin, transparent membrane. When the lesion occurs early in pregnancy, it may heal before delivery leaving a congenital atrophic alopecic scar. The non-scalp ACC are usually of large size and may be associated with of epidermolysis bullosa (EB).[6] The association of ACC with EB may be the visible sign of other congenital anomalies such as pyloric or duodenal atresia, ureteral stenosis, renal abnormalities, craniofacial abnormalities and nail dystrophy.[7,8] Histological examination of the lesion shows the absence of normal skin structures such as hair follicles, sebaceous glands or sweat glands with the dermis lacking of collagen fibres.

ACC was reported to affect 1 in every 10 000 live births.[9] Another review in 2006 reported an incidence of 2.8 cases per 10 000 newborns.[10] This apparent discrepancy in the incidence may be the result of the significant underreporting.

Many theories have been postulated to explain the occurrence of ACC; however, neither the pathogenesis nor the aetiology has been clarified yet. Factors like intrauterine trauma, amniotic bands and drugs such as methimazole have been implicated.[9,11] Most published cases of ACC are sporadic; with a few reports describing a familial occurrence in the form of autosomal dominant[12], as well as autosomal recessive[13] pattern of inheritance.

## CASE REPORTS

### Case 1

**Figure 1:**
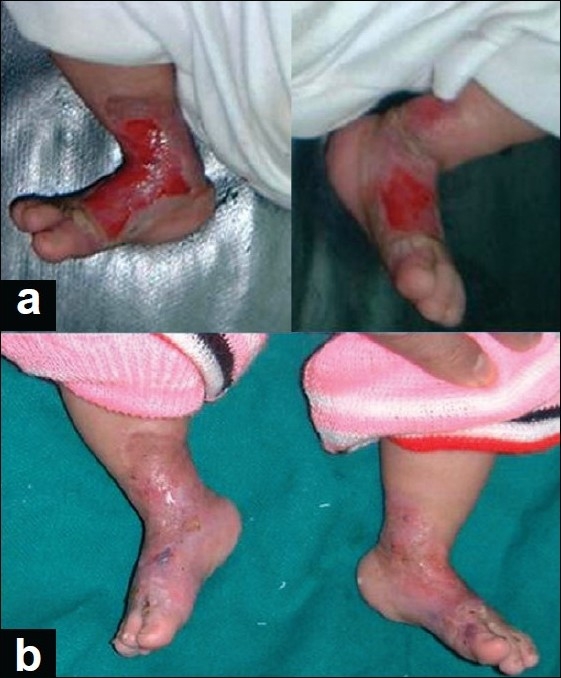
Case 1; Bilaterally-symmetrical involvement of the ankle regions of the baby with the peripheral healing already begun when the child was presented by his 10th postbirth day (a) with complete healing after 37 days of treatment (b)

A 10-day old male baby was referred to us with bilateral symmetrical ulcers involving the areas of ankle joints with the adjacent portions of the legs and feet. Healing was in progress from the peripheries and was completed in the next 27 days. The last parts to heal were the skin over the medial malleolus and anterior aspect of the ankle joint [Figure 1].

### Case 2

**Figure 2:**
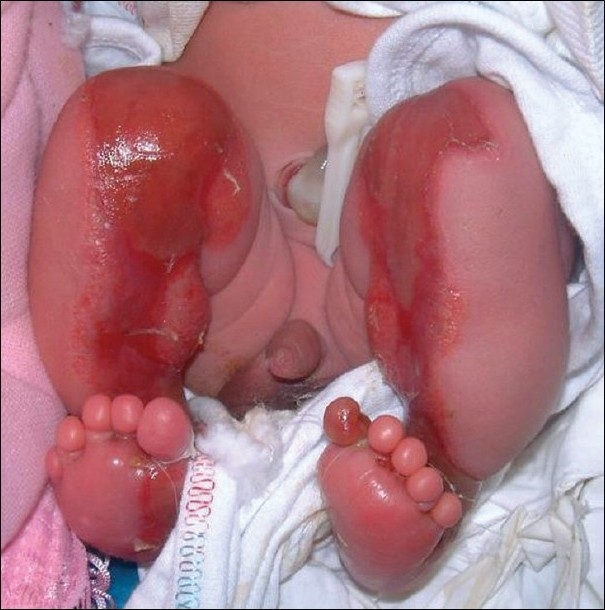
Case 2; A newborn with bilateral symmetrically-distributed aplasia cutis involving the antero-medial aspects of both lower limbs with involvement of the medial third of the right sole.

**Figure 3:**
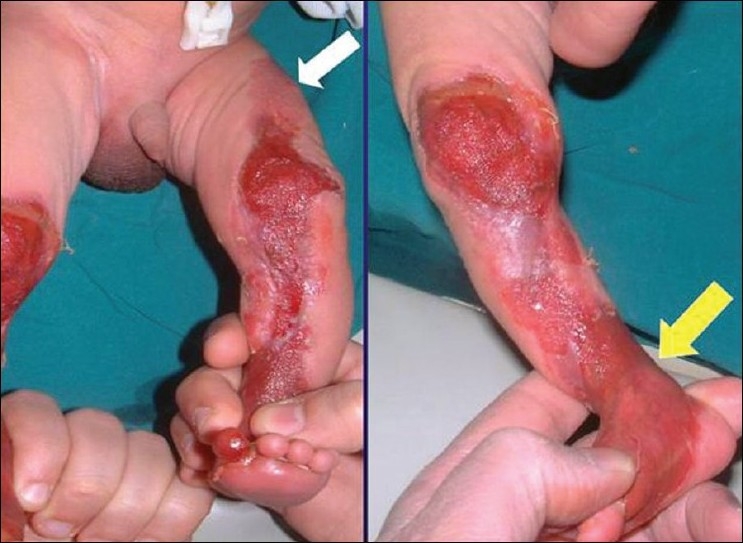
The upper part is already healed and is presented as a congenital scar (white arrow) and the lower area is covered by the thin translucent membrane ‘yellow arrow’

A 2-day old baby was referred by an obstetrician for extensive areas of denuded skin in both lower limbs [Figure 2]. The full term child, born by normal delivery, had bilaterally symmetrical distributed aplasia cutis involving the antero-medial aspects of both lower limbs with involvement of the medial third of the right sole No family history of similar condition and no history of medications or disease during pregnancy could be elicited. On physical examination, the child showed no signs of acute distress or neurological impairment. The uppermost part in the left thigh had already healed and is presented as a congenital scar [Figure 3] and the lower area was covered by the thin translucent membrane ‘yellow arroa’). Histological examination of a punch biopsy taken from the lesion showed an absence of epidermis and adnexal structures.

**Figure 4:**
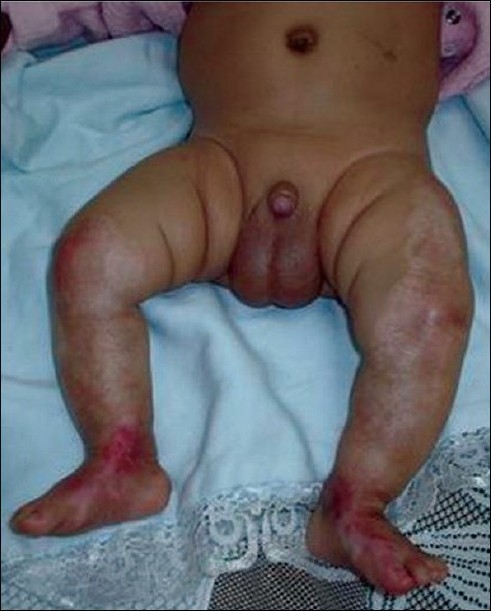
Healing was complete by the 45th day of treatment with residual pigmentory changes in the scar.

The healing process was evidently rapid in the first 2 weeks of treatment especially in the areas away from the joints and complere healing occured in 45 days. [Figure 4].

### Management

Both neonates were treated conservatively. This consisted of gentle cleansing of the denuded area with saline-diluted bovidine iodine lotion and then covering it with antibiotic-impregnated non-adherent dressing to promote healing in a moist environment. The dressing was changed every 5-7 days.

## DISCUSSION

Few conditions may be associated with ulceration in the newborn. The most common lesion of them is the ACC either alone or with EB. In transient bullous dermolysis, which is a form of dystrophic EB, the baby may have blisters in the limbs.[14] Congenital herpes may rarely be the cause of congenital abrasions.[15] Neonate with Setleis syndrome may have depressed scarred areas on the temporal scalp resembling healed ACC; however, it can be differentiated easily by its characteristic facial features especially periorbital puffiness and inverted V-shaped mouth.[16] Other causes of ulcerations in neonates that should be differentiated from ACC include scalp electrode[17] and pyoderma gangrenosum.[18]

The management of non-scalp ACC is still controversial. Most lesions heal spontaneously with conservative dressing, but large lesions may necessitate surgical interference with skin grafts or local skin flaps.[2] Fresh allograft has been used as temporary biological dressing to enhance epithelization of the defects.[19] Cultured epithelial autografts have been used together with acellular allogenic dermal grafts.[20] Skin grafting is limited by donor-site availability, potential morbidity and the technical difficulties associated with handling the thin neonatal skin. Flap reconstruction involves subjecting a neonate to anaesthesia and a major surgical procedure, with the risk of significant blood loss. Although the use of cultured keratinocytes is promising, it is still restricted to centres having tissue culture laboratory.

In our two cases, the relatively large wounds were managed conservatively on an outpatient basis and in the two cases; healing was complete in an average period of 41 days without the need for hospitalization. Domiciliary treatment allowed normal breast-feeding of the neonates without disturbing or interfering with his parents' life. Epithelization was noticed first in the areas away from the joints; this may be due to the effects of joint mobility on the epithelization process. We did not use slabs to immobilize the nearby joints; instead we used bulky cotton padding that limited the joint mobility rather than completely abolishing it.

Conservative outpatient management of the ACC is simple and it obviates the stress of hospital stay and the possibility of infections by resistant hospital borne micro-organisms. Although the wound healed by secondary intention, this spontaneous healing in our patients did not result in any significant scarring or contractures in the nearby joints. However, there was slight abnormal hypopigmentation in the healed skin.
